# Multiple novel hepatocellular carcinoma signature genes are commonly controlled by the master pluripotency factor OCT4

**DOI:** 10.1007/s13402-019-00487-3

**Published:** 2019-12-17

**Authors:** Chao Ye, Xiaoqian Zhang, Xinyu Chen, Qingyi Cao, Xiaobing Zhang, Yanwen Zhou, Wenxin Li, Liangjie Hong, Haiyang Xie, Xiaoli Liu, Hongcui Cao, Ying-Jie Wang, Bo Kang

**Affiliations:** 1 State Key Laboratory for Diagnosis and Treatment of Infectious Diseases, Collaborative Innovation Center for Diagnosis and Treatment of Infectious Diseases, the First Affiliated Hospital, School of Medicine, Zhejiang University, Zhejiang, 310003 Hangzhou China; 2 The First Affiliated Hospital of USTC, Division of Life Sciences and Medicine, University of Science and Technology of China, Hefei, 230001 Anhui China; 3 Department of Infectious Diseases, the Second Xiangya Hospital, Central South University, Changsha, 410011 Hunan China; 4 Department of Respiratory Medicine, Institute of Respiratory Disease, The First College of Clinical Medical Sciences, Yichang Central People’s Hospital, China Three Gorges University, Yichang, 443003 Hubei China; 5 NHFPC Key Laboratory of Combined Multi-organ Transplantation, the First Affiliated Hospital, School of Medicine, Zhejiang University, Hangzhou, 310003 China; 6 Key Laboratory of the Diagnosis and Treatment of Organ Transplantation, CAMS, the First Affiliated Hospital, School of Medicine, Zhejiang University, Hangzhou, 310003 China

**Keywords:** Hepatocellular carcinoma, OCT4, Biomarker, Therapeutic target, Transcriptional regulation, RNA sequencing

## Abstract

**Abstract:**

**Background:**

Worldwide, hepatocellular carcinoma (HCC) is a common solid tumor with a poor prognosis. HCC is often due to hepatitis B virus (HBV) infection. As yet, efficacious HCC treatment regimens for late-stage HCC patients are lacking. Therefore, the identification of more specific and sensitive biomarkers for its early diagnosis and treatment remains an urgent need.

**Methods:**

Total RNAs from paired HBV-derived HCC tumors and adjacent peritumor tissues (APTs) were subjected to RNA sequencing (RNA-seq), and differentially expressed genes (DEGs) between HCC tumors and APTs were selected and verified.

**Results:**

We identified 166 DEGs and found that eight top-ranked and verified DEGs (*TK1*, *CTTN*, *CEP72*, *TRIP13*, *FTH1*, *FLAD1*, *CHRM2*, *AMBP*) all contained putative OCT4 binding motifs in their promoter regions. TK1, TRIP13 and OCT4 were found to exhibit concurrent higher expression levels in HCC tumors than in APTs. The mRNA levels of TK1, TRIP13 and OCT4 in a cohort of 384 HCC samples from the TCGA database were all found to be negatively correlated with patient overall survival, relapse-free survival and progression-free survival, underscoring the HCC biomarker status of TK1 and TRIP13 on one hand, and implicating their association with OCT4 on the other hand. Furthermore, OCT4 proteins were found to bind to the promoters of both genes in vitro and in vivo. Knocking out *OCT4* in HCC-derived cell lines reduced the expression of *TK1* and *TRIP13* and significantly decreased their tumorigenicity.

**Conclusions:**

Using RNA-seq, we identified several novel HCC signature genes that may serve as biomarkers for its diagnosis and prognosis. Their common transcriptional regulation by OCT4 suggests key roles in the development of HCC, and indicates that OCT4 may serve as a potential therapeutic target.

**Electronic supplementary material:**

The online version of this article (10.1007/s13402-019-00487-3) contains supplementary material, which is available to authorized users.

## Introduction

Hepatocellular carcinoma (HCC) is a common solid tumor and the third leading cause of cancer-related death worldwide (second leading cause of cancer-related death in less developed countries) [1]. Every year, ~500,000 new cases are diagnosed in the Asia-Pacific region, with more than 60% of the total number occurring in China alone, often due to hepatitis B virus (HBV) infection [2]. Individuals with chronic HBV infections, especially those with chronic liver disease and cirrhosis, are at an increased risk of developing HCC [3, 4]. Compared to HCCs associated with other risk factors, HBV-related HCCs exhibit higher rates of chromosomal alterations and p53 mutations, enhanced activation of certain signaling pathways (e.g. the WNT/β-catenin pathway) and elevated expression levels of fetal liver/hepatic progenitor genes [5].

As highly efficacious treatment regimens for late-stage HCC patients are still lacking, early diagnosis and intervention remain the key to improve survival. Several invasive and non-invasive diagnostic biomarkers [e.g. α-fetoprotein (AFP), AFP-L3, des-γ-carboxyprothrombin (DCP)] have already been identified and evaluated in different clinical settings [6]. However, those biomarkers display limited sensitivity and specificity, especially with respect to early HCC stages and, therefore, combinations with other newly-identified candidate biomarkers are currently being evaluated [6]. Over the past years, technologies including whole genome sequencing (WGS), RNA-sequencing (RNA-seq) and proteomic profiling have led to a new era in biomarker development that has improved our understanding of complex interactions between proteins, genes and noncoding RNAs in hepatocarcinogenesis in different settings [7–9]. Most studies did, however, not distinguish HBV-related HCCs from HCCs resulting from other factors, and the analyzed tumor tissues were rarely matched with adjacent peritumor tissues (APTs) from the same patients. Thus far, only two RNA-seq-based genome-wide transcriptome analyses have been reported, identifying HBV-related HCC biomarkers using matched patient samples. Huang et al. [10] for the first time conducted genome-wide transcriptome analyses of 10 matched pairs of cancer and non-cancerous tissues from HCC patients. They found that the 1378 differentially expressed genes (DEGs) identified were mostly enriched in 54 bio-function terms and 41 canonical pathways, thereby providing important clues for our understanding the molecular mechanisms underlying HCC development. In a subsequent study, Miao et al. [11] conducted comparative multi-omics profiling of a complete collection of representative HCC patient samples for HCC biomarker identification. Four DEGs (*SFN*, *TTK*, *BUB1*, *MCM4*) were found to be associated with different tumor differentiation patterns, and the dual-specificity protein kinase TTK was identified as a promising prognostic biomarker for HBV-related HCC. However, an in-depth functional characterization of the DEGs or candidate biomarkers was lacking in the above two studies.

Here, we used RNA-seq to decipher and compare whole transcriptomes of paired tumor tissues and APTs from three HBV-related HCC patients and, by doing so, generated a list of DEGs. The most highly-ranked DEGs were further verified as potential HCC signature genes using qRT-PCR in another independent cohort of 30 HCC patients. Remarkably, we found that eight out of eight top-ranked and validated DEGs (*TK1*, *CTTN*, *CEP72*, *TRIP13*, *FTH1*, *FLAD1*, *CHRM2*, *AMBP*) contained putative OCT4 binding motifs in their promoter regions, and two of them (*TK1*, *TRIP13*) were verified as direct transcriptional targets of the master pluripotency factor OCT4.

## Materials and methods

### Reagents

Monoclonal (sc-5279) and polyclonal (ab19857, ChIP grade) anti-OCT4 antibodies were purchased from Santa Cruz Biotechnology and Abcam, respectively. Monoclonal anti-TK1 (ab-76,495) and polyclonal anti-TRIP13 (ab-204,331) antibodies were purchased from Abcam. Peroxidase-conjugated anti-mouse secondary antibody (7076), peroxidase-conjugated anti-rabbit secondary antibody (7074), normal Rabbit IgG (2729) and a biotinylated protein ladder detection pack (7727) were all purchased from Cell Signaling Technology. An anti-GAPDH (AG019) antibody was purchased from the Beyotime Institute of Biotechnology.

### Patients and specimens

The source of the HCC patient samples has been described before [12]. Briefly, 33 HCC patient samples were included for RNA-seq and validation in this study; samples from three male HBV-related HCC patients (T1/P1, T2/P2, T3/P3) were used for RNA-seq. Tumor tissues and paired APTs were collected at the time of hepatic carcinectomy. The tissues were collected strictly within the boundaries of tumors, whereas the APTs were collected at least 3 cm away from the tumor margins. All collections were conducted under supervision of the same pathologist. Pathological diagnoses were conducted by two independent and expert pathologists. All tumor tissues were confirmed as primary hepatocellular carcinoma. Retrospective data including demographic, preoperative laboratory and pathologic parameters were collected from electronic medical records. The 33 HCC patient cohort encompasses 27 men and 6 women, with a mean age of 56. All 33 patients were HBV surface antigen-positive without hepatitis C virus (HCV) infection and exhibited the same underlying cirrhosis etiology. Clinical information of these patients is listed in Table 1.

**Table 1:** Demographic and laboratory parameters of the subjects included in the study

Samples	RNA-seq cohort			Validation cohort
	T1/P1	T2/P2	T3/P3	*N* = 30
Demographic parameters				
Gender (M/F)	M	M	M	24/6
Age (y) (x ± SD)	52	39	57	51.69 ± 6.69
Laboratory parameters [median (range)]				
ALT (U/L)	30	54	34	21.25 (12.0–170.0)
AST (U/L)	108	43	40	62.5 (20.0–355.0)
TBiL (μmol/L)	12.1	24.4	11.6	49.9 (5.0–384.0)
AKP (U/L)	63	58	104	106.25 (47.0–349.0)
GGT (U/L)	301	126	69	122.75 (14.0–591.0)
Pathologic parameters				
AFP (ng/ml)	> 50,000	12,628.5	8214	2401.1 (1.5- > 50,000)
CEA (ng/ml)	2.3	2.0	2.8	1.97 ± 0.79
CA199 (U/ml)	39.3	37.6	6.1	14.95 (2.2–212.3)
CA125 (U/ml)	6.1	7.4	4.7	121.58 (5.6–208.1)
Ferritin (ng/ml)	534.2	413.9	567.1	11,229.45 (43.5- > 40,000)
Tumor				
Number (S/M*)	M	S	S	14/16
Size (cm)	9	3.5	8.5	4.65 (1.5–25.0)
Histopathologic grading (poor/ moderate/high)	poor	moderate	moderate	16/10/4
PVTT* (Yes/No)	YES	YES	YES	4/26

S/M* single/multiple, PVTT* Portal vein tumor thrombus

### Cell lines and culture

The human HCC lines Huh7 and Hep3B were purchased from the Cell Bank of Type Culture Collection of the Chinese Academy of Sciences (Shanghai, China) and cultured in Dulbecco’s modified Eagle’s medium (DMEM) containing 10% fetal bovine serum (FBS) (both from Hyclone^R^ GE, USA) under humidified conditions at 37 °C with 5% CO_2_.

### RNA library preparation and sequencing

Total RNAs of HCC tumor tissues and paired APTs from three HBV-related HCC patients were isolated using Trizol reagent (Lift Technologies, USA). RNA-library preparation was conducted using an Illumina standard kit according to the manufacturer’s protocol. Briefly, poly-A containing RNAs were purified, followed by fragmentation into small pieces. Next, the RNA fragments were converted into single-strand cDNA using superscript II reverse transcriptase (Invitrogen) and random hexa-primers (IDT, Coralville, Iowa, USA), followed by second strand synthesis using DNA polymerase I (Invitrogen) and *E. coli* RNase H (Invitrogen). After second strand synthesis, with end repairing and A-tailing, the synthesized double-stranded cDNA fragments were subjected to purification and subsequently ligated to Illumina adapters using a Quick ligation TM kit (NEB) and DNA ligase. The resultant cDNA adapter-modified cDNA libraries were fractionated on agarose gels, after which 200-bp fragments were excised and amplified by 15 polymerase chain reaction (PCR) cycles. After purification, the quality of the cDNA libraries was checked using a Bioanalyzer 2100 (Agilent). Next, the concentrations of the cDNA libraries were measured and diluted to 10 nM in Tris-HCl buffer prior to cluster generation. Cluster formation, primer hybridization and sequencing reactions were performed sequentially according to the manufacturer’s recommended protocol. The sequencing procedure was conducted on an Illumina® Hiseq2000 apparatus.

TopHat software [13] was used to map reads complying with quality standards to the human reference genome assembly hg19 (NCBI build: GRCh37) with default parameters. The coordinates of the mapped reads were overlaid with genomic coordinates of a human gene set defined in RefSeq (NCBI) and then counted to determine the expression level of each individual gene. The expression level for each gene was then normalized to reads per kilo base of transcript per million mapped reads (RPKM). Differential expression analysis of each gene between tumor tissues and APTs was performed using the Limma package of Bioconductor from raw read counts. The false discovery rate (FDR) of each gene was determined according to the Benjamini–Hochberg procedure and a mean log2 (fold change [RPKM of tumor/ RPKM of APTs]) was calculated across all genes. The significantly differentially expressed genes (DEGs) were selected with their FDRs < 0.05 and fold change > 2 between tumors and APTs.

### Functional enrichment analysis of DEGs

Functional enrichment analysis was employed to roughly characterize the DEGs in HCC tumorigenesis. Gene ontology (GO) (web-based gene set analysis tool kit) is a standard classification system of gene function and gene products. In addition, PANTHER (http://go.pantherdb.org/) and Kyoto Encyclopedia of Genes and Genomes (KEGG) (http://www.kegg.jp/) pathway analyses were used to reveal the potential roles of DEGs in liver carcinogenesis.

### RNA isolation and DEG validation

The selected DEGs were initially validated by quantitative real-time PCR (qRT-PCR) using the same RNA samples as those for RNA sequencing. Furthermore, total RNAs were extracted from tumor tissues and paired APTs of 30 independent HBV-related HCC patients using an RNeasy Mini Kit (Qiagen, the Netherlands). The concentration and quality of the RNAs were determined using Merinton SMA1000. The cDNAs were synthesized using a Prime Script^Tm^ RT Master Mix (TaKaRa, Japan) according to the product manual. qRT-PCR of DEGs was performed using SYBR Premix Ex TaqII (TaKaRa, Japan) and analyzed on an ABI system 7500 (Life Technologies, USA). All assays were carried out independently in triplicate. The *GAPDH* or *B2M* genes were used as references for quantification. Relative gene expression values, expressed as fold changes, were subsequently determined using the Delta-Delta Ct method. The primers used for qRT-PCR are listed in Table S1.

### Analyses of associations between HCC signature genes and HCC clinical outcomes

We retrieved mRNA expression levels of eight HCC signature genes and *POU5F1* from The Cancer Genome Atlas (TCGA) RNA sequence database (https://genome-cancer.ucsc.edu/). Patients meeting the following criteria were included in this study: pathological diagnosis with HCC and the availability of overall survival (OS), relapse-free survival (RFS), progression-free survival (PFS) and clinicopathological information. After excluding incomplete clinical data obtained from TCGA dataset, 348 cases were grouped and characterized (Table S2). Survival curves for HCC patients were generated using an online database, Kaplan-Meier plotter (http://kmplot.com/analysis/). The above-mentioned 384 patients were divided into high-expression and low-expression groups using median values of mRNA expression. Statistical differences in survival were assessed by log-rank (Mantel-Haenszel) test, and *p* < 0.05 was considered statistically significant. Hazard ratios (HR) and *p*-values were calculated online.

### CRISPR/Cas9 knockout assay

OCT4 knockout (OCT4-KO) Huh7 or Hep3B cells were established using a CRISPR/Cas9 lentiviral system according to a standard protocol from Feng Zhang’s laboratory. In brief, single guide RNA (sgRNA) targeting OCT4A was generated as described previously [14] and cloned into a pLentiCRISPRv2 vector. After confirming the sgRNA efficiency, we prepared OCT4-KO and non-targeting control (NTC) lentiviruses by transfecting the lentiCRISPRv2-KO and NTC, psPAX2, pMD2.G plasmids into 293 T cells, respectively, after which the virus titers were functionally determined and used to infect Huh7 or Hep3B cells at a low multiplicity of infection (MOI; 0.3–0.5). The infected cells were selected under 0.5–1 μg/ml puromycin for 7 days, and expanded for another 7 days for Surveyor assay, qRT-PCR and further functional characterizations.

### Western blotting

Protein concentrations of tumor tissues and APTs were quantified using a Pierce™ BCA Protein Assay Kit (Thermo Fisher Scientific 23,227). Next, the samples were boiled for 30 min and the supernatants were loaded for SDS-PAGE (Bio-Rad) and transferred to PVDF membranes (Bio-Rad), which were incubated sequentially with primary and secondary antibodies, and developed using ECL reagent as described previously [15]. GAPDH was used as an internal control for sample loading.

### Immunohistochemistry

Immunohistochemical (IHC) examination of formalin-fixed tumor tissue sections was conducted as previously described [16] using anti-TK1 (diluted at 1:100), anti-TRIP13 (diluted at 1:100) or anti-OCT4 (diluted at 1:500) antibodies.

### Electrophoretic mobility shift assay (EMSA)

EMSA was conducted using a LightShift chemiluminescent EMSA kit (Thermo 20,148) according to the manufacturer’s instructions. Cell nuclear extracts were prepared using NE-PER™ Nuclear and Cytoplasmic Extraction Reagents (Thermo Fisher Scientific 78,833). Subsequently, EMSA was performed as described previously [17]. The sequences of double-strand biotin-labeled *TK1* and *TRIP13* probes (with the putative OCT4 motifs being underlined) were as follows.

*TK1* probe 1: Biotin-5′GGGACCTGGCACGCACTAGGCGCTCTGCATGCCCACAGGAGTGCTCTAGACG 3′.

- Biotin-5′CGTCTAGAGCACTCCTGTGGGCATGCAGAGCGCCTAGTGCGTGCCAGGTCCC 3′.

*TK1* probe 2: Biotin-5′CCTGGCAGGGTCTACGGATATTATTAGCATAGTCAGGACTTCAATTTTCTTT 3′.

- Biotin-5′AAAGAAAATTGAAGTCCTGACTATGCTAATAATATCCGTAGACCCTGCCAGG 3′.

*TK1* probe 3: Biotin-5′CGGGCTAACACCTTCACACTTTATGCAGAAAGTAACAAGGAACCATTCTGAG 3′.

- Biotin-5′CTCAGAATGGTTCCTTGTTACTTTCTGCATAAAGTGTGAAGGTGTTAGCCCG 3′.

*TRIP13* probe: Biotin 5′GGGAATTACCTGCGTTTTCACTGACATGCATCTCTCTTACCAGTCTGACCCAGATGGGG 3′.

- Biotin 5′CCCCATCTGGGTCAGACTGGTAAGAGAGATGCATGTCAGTGAAAACGCAGGTAATTCCC 3′.

### Chromatin immunoprecipitation (ChIP)

ChIP analysis was performed as described previously [17] using an EZ-ChIP™ Chromatin Immunoprecipitation Kit (Merck Millipore 2,673,061). Briefly, 2 × 10^7^ - 5 × 10^7^ cells were chemically cross-linked by the addition of 1% formaldehyde solution for 10 min at room temperature. The reaction was stopped by adding glycine to a final concentration of 125 mM. Next, he cells were rinsed twice with cold PBS and harvested using a silicon scraper. The resulting cell samples were sonicated to solubilize and shear cross-linked DNA to an average size of 300–800 bp. Immunoprecipitation was carried out using 5 μg rabbit anti-OCT4 (ab 19,857) and 100 μl protein A/G agarose beads (Pierce 20,421) (with normal rabbit IgG being the negative control). The primers of *TK1* and *TRIP13* for amplifying the DNA fragments were as follows: *TK1*: F 5’-CTGGCAGGGTCTACGGATAT-3′ and R 5′ -CCGTCTAGAGCACTCCTGT-3′. *TRIP13*: F 5′- TCGAGGTCCCTTCTTCCCAA-3′ and R 5′- AGTAGCCCCATCTGGGTCAG-3′. ChIP-real time PCR was performed using SYBR Premix Ex TaqII (TaKaRa, Japan) and analyzed on an ABI system 7500 (Life Technologies, USA). Extracted DNA fragments and input genomic DNAs served as templates. The anti-OCT4 precipitated DNA fragments corresponding to specific genes were quantified using qPCR and expressed as fold enrichment over anti-IgG precipitated DNA fragments. The Delta-Delta Ct method was used for relative quantification.

### Mouse xenograft tumor model

NOD/SCID mice (female, 3–4 weeks old) were purchased from the Shanghai Experimental Animal Centre, Chinese Academy of Science. They were kept in the central animal facility of the First Affiliated Hospital of School of Medicine, Zhejiang University and housed in laminar-flow cabinets under specific pathogen-free conditions with a 12 h light/dark cycle. All studies on mice were conducted in accordance with the National Institute Guide for the Care and Use of Laboratory Animals. The animal protocol has been approved by the Committee of the Ethics of Animal Experiments, Zhejiang University.

For subcutaneous xenografting experiments, the mice were randomly divided into a treatment group (OCT4-KO) and a control group (NTC), and OCT4-KO or NTC Huh7 cells (2 × 10^6^) were inoculated subcutaneously into each mouse. The diameters of the tumors were measured every three days with precision calipers. The tumor mass (xenograft) volumes were calculated using the formula: volume = [(tumor length) × (tumor width)^2^]/2. At day 18 after inoculation, the mice were sacrificed and the tumors removed, weighed and photographed.

### Statistical analysis

All continuous variables were expressed as mean ± standard deviation (S.D.) or medians, and interquartile ranges. Comparisons of continuous variables were performed using Student *t* test or nonparametric Mann-Whitney U test. Comparisons between paired groups were carried out using paired t test or Wilcoxon signed ranks test when necessary. Correlations between RNA-seq measures and qRT-PCR measures were determined using the Spearman rank correlation coefficient. All data analyses were performed using SPSS 20.0 (Chicago, IL).

## Results

### Identification and preliminary characterization of DEGs

RNA-seq was performed using three pairs of matched HBV-related HCC tumors and APTs, and the overall characteristics of the RNA-seq data are summarized in Table S3. No significant differences were observed between the numbers of genes identified in each tumor and APT pair. An average of 12,073 and 8095 genes was found to be up-regulated or down-regulated, respectively, in tumor tissues compared with paired APTs (Table S4). The coverage levels of all genes sequenced in each sample are shown in Fig. S1. The Q30 value of every library was greater than 80%. Evaluation of sequencing randomness and saturation showed that the RNA-seq data are good in quality.

**Fig. 1:**
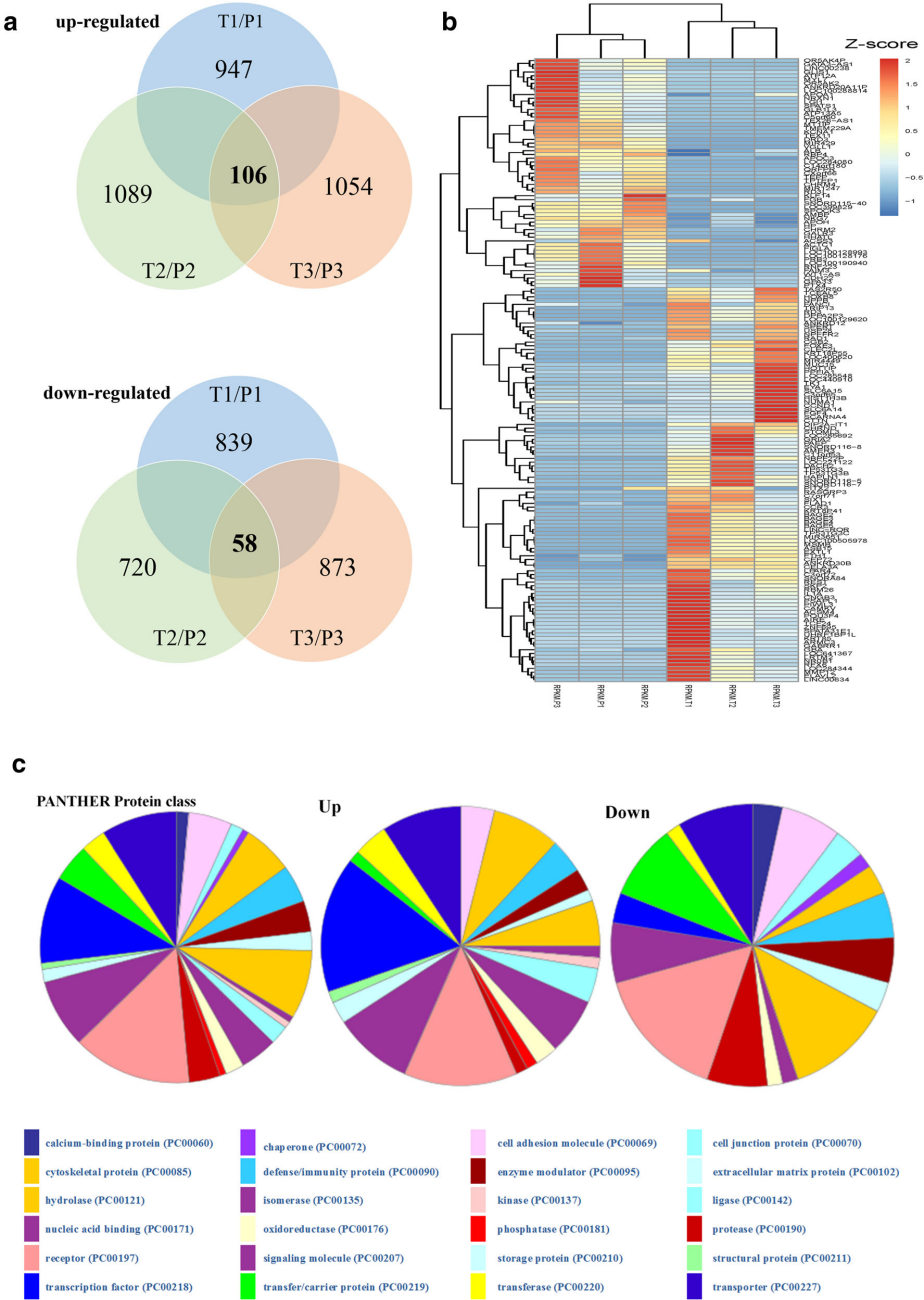
Identification and preliminary characterization of DEGs in three paired HCC tumor tissues (T) and adjacent peritumor tissues (P). **a** Composition of DEGs in three paired samples used for RNA-seq. **b** Heat map generated from normalized FPKM of 166 consistent DEGs across three paired samples. The expression level of each transcript is represented by a color, ranging from blue (low) to red (high). **c** PANTHER protein class categories of total DEGs, and increased and decreased DEGs in pie charts. The chart legends provide detailed information on protein class categories

Based on the DEG selection criterion (i.e., FDRs < 0.05 and fold change > 2 between each paired tumor and APT), the expression levels of 947, 1089 and 1054 genes were up-regulated in the tumor tissues (T1, T2, T3) compared to the paired APTs (P1, P2, P3), respectively. Meanwhile, the expression levels of 839, 720 and 873 genes in the corresponding samples were down-regulated (Fig. 1a). Among all these DEGs, there were 105 up-regulated and 61 down-regulated DEGs commonly identified in all three paired HCC samples. The relative expression levels of these 166 DEGs are shown in a heat map (Fig. 1b).

Next, functional enrichment analyses were employed to initially characterize the above DEGs. Based on gene ontology (GO) analysis, 38 terms encompassing 23 of biological process (BP), 14 of cellular compound (CC) and 1 of molecular function (MF) were significantly over-represented among all DEGs (adjusted *p* value < 0.05) (Table S5). Based on gene/protein classification at the PANTHER website, the up-regulated genes in the tumor tissues were mainly associated with “transcription factor and signaling molecule”, while the genes with decreased expression in the tumor tissues were mainly associated with “receptor and hydrolase” (Fig. 1c). Based on KEGG pathway analysis, significantly enriched KEGG pathways of the up-regulated genes included “cell cycle” and “pathway in cancer”.

### Validation of top-ranked DEGs as HCC signature genes

**Fig. 2:**
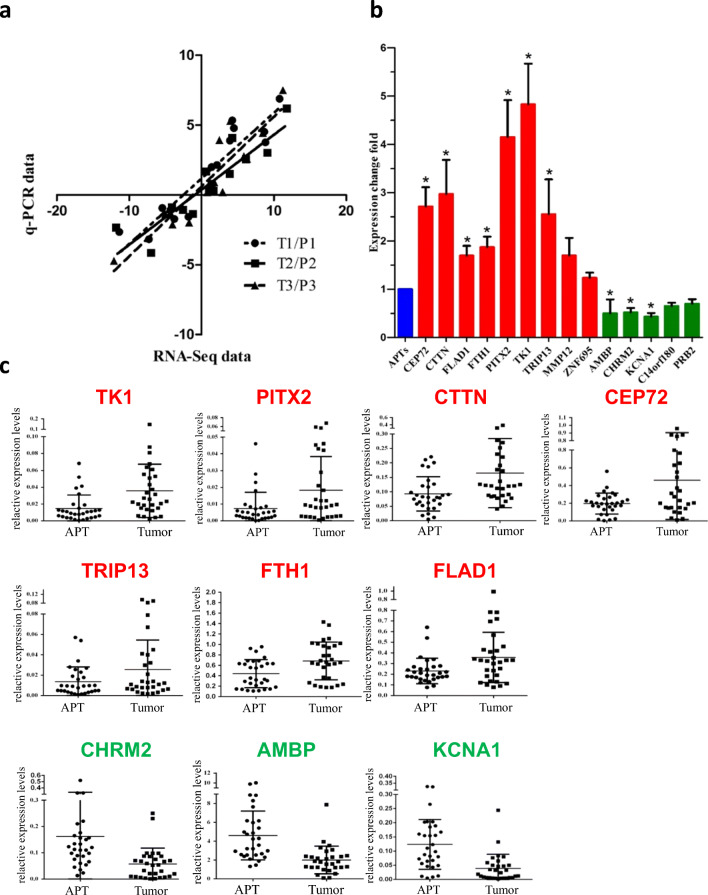
Validation of top-ranked DEGs as HCC signature genes. **a** Correlation between RNA-seq and qRT-PCR data of 14 selected genes in 3 pairs of amplified RNA samples. Spearman rank correlation coefficient = 0.83, *p* < 0.01. **b** Expression levels of DEGs in validation cohort, including up-regulated (red) and down-regulated (green) levels in tumor tissues. Expression levels in APTs (blue) were used as a reference. *p < 0.05. **c** Expression levels of 10 DEGs in paired HCC tumor tissues (Tumor) and adjacent peritumor tissues (APT), respectively

To validate the reliability of the RNA-seq data, 14 out of 166 highly ranked DEGs were selected and analyzed for their mRNA levels by qRT-PCR using the original six amplified RNA samples for RNA-seq. These top-ranked DEGs included 9 up-regulated genes (*TK1*, *PITX2*, *CTTN*, *CEP72*, *TRIP13*, *FTH1*, *FLAD1*, *MMP12*, *ZNF695*) and 5 down-regulated genes (*CHRM2*, *AMBP*, *KCNA1*, *C14orf180*, *PRB2*). Log2 fold changes of qRT-PCR measurements were compared with those of RNA-seq measurements for the 14 selected DEGs, and their correlation coefficient reached 0.82 (Fig. 2a). Hence, we conclude that our RNA-seq method could reliably measure gene expression differences in HCC samples. Next, qRT-PCR validation was extended to a larger number of samples from another independent cohort including 30 patients diagnosed as HBV-related HCC. The laboratory parameters of 24 male and 6 female patients with an average age of 51.69 years are presented in Table 1. Fourteen of them had a single HCC tumor and 16 had multiple tumors. Most of the tumors were poorly or moderately differentiated. Four of them exhibited portal vein tumor thrombus (PVTT).

We found that 10 out of 14 DEGs showed consistent expression differences in the validation cohort (Fig. 2b). Among them, the mRNA levels of *TK1*, *PITX2*, *CTTN*, *CEP72*, *TRIP13*, *FTH1* and *FLAD1* were consistently and significantly up-regulated in the tumor tissues compared to the APTs, while those of *CHRM2*, *AMBP* and *KCNA1* were significantly down-regulated in the tumor tissues. Fig. 2c shows the expression levels of each HCC signature gene in paired HCC tissues and APTs. To additionally compare our RNA-seq data with corresponding data available in the literature, we retrieved the supplementary data reported by Huang et al. [10] who performed genome-wide RNA-seq analyses of 10 matched cancer and non-cancerous tissue pairs of HBV-related HCC patients. The reads per kilobase of transcript per million mapped reads (RPKM) values of 10 matched samples were selected for the above 10 DEGs (Table S6). The tumor/peritumor ratios (T/P) of 10 pairs of matched samples were calculated and plotted as fold changes of expression levels of the DEGs (Fig. S2). Since the RPKM values of two DEGs (*PITX2*, *KCNA1*) were too small to be considered, we eliminated these two hereafter. Pairwise comparisons between the tumor versus peritumor samples for each DEG are presented in Fig. S3. In addition, we compared the dataset of Huang et al. (Fig. S2) with that of our own (Fig. 2b), and found that the correlation coefficient of these two sets of RNA-seq data was 0.882, indicating a high effectiveness of the experimental systems in both studies. Taken together, we conclude that eight DEGs (*TK1*, *CTTN*, *CEP72*, *TRIP13*, *FTH1*, *FLAD1*, *CHRM2*, *AMBP*) may be considered as novel HBV-related HCC signature genes.

### Identification of OCT4 as a common upstream regulator for HCC signature genes

**Fig. 3:**
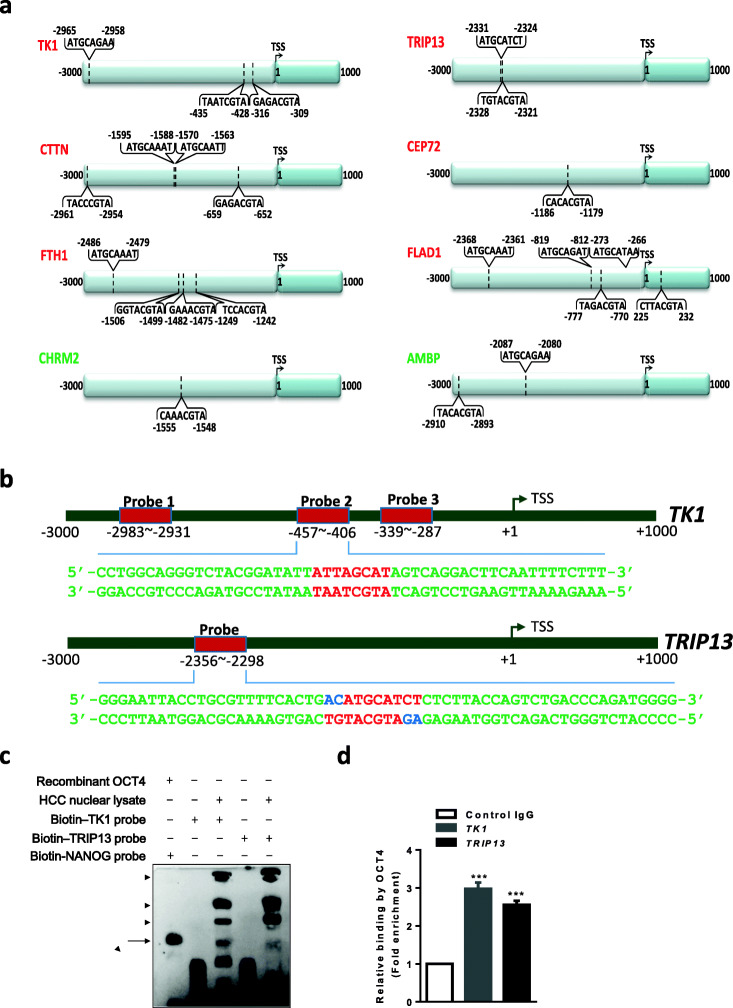
OCT4 binds to the *TK1* and *TRIP13* gene promoters. **a** OCT4 emerged as only transcription factor with a canonical binding motif (ATGCA/TAAT) in all 8 HCC signature genes. Three of them (CEP72, TRIP13, CHRM2) had a single OCT4 binding motif, while the others had multiple OCT4 binding motifs in their promoter regions. **b** The position of EMSA probes (red boxes) relative to the transcription start sites (TSS) of the *TK1* and *TRIP13* genes. Also shown are partial promoter sequences of the *TK1* and *TRIP13* genes that span the putative OCT4-binding motifs (highlighted in red). **c** EMSA of biotinylated *TK1* or *TRIP13* probes incubated with nuclear extracts of Hep3B cells, and as a positive control EMSA of biotinylated *NANOG* probes, incubated with recombinant human OCT4 proteins. The arrow indicates complexes formed between the biotinylated probes and monomeric OCT4 proteins, while the arrowheads indicate the biotinylated probes bound with larger protein complexes including OCT4. **d** 2 × 10^7^– 5 × 10^7^ Hep3B cells were chemically cross-linked by 1% formaldehyde for 10 min at room temperature, after which the cell samples were sonicated to solubilize and shear cross-linked DNA to an average size of 300–800 bp. Immunoprecipitation was carried out with 5 μg rabbit anti-OCT4 (ab 19,857) and 100 μl protein A/G agarose beads (Pierce 20,421), with normal rabbit IgG serving as negative control. The anti-OCT4 precipitated DNA fragments corresponding to the *TK1* or *TRIP13* genes were quantified using qPCR and expressed as fold enrichment over anti-rabbit IgG precipitated DNA fragments (Control IgG). The data are expressed as mean ± S.D. of triplicate measurements from one of three independent experiments, which yielded similar results. ***p < 0.001

To gain insight into potential regulatory mechanisms for the above identified HCC signature genes, a 4 kb (−3 kb to +1 kb relative to TSS) promoter region for each HCC signature gene was analyzed by Genomatix for the presence of potential transcription factor binding sites. Despite a number of transcription factors that were predicted to be differentially or partially commonly associated with the promoters of the eight genes, OCT4 emerged as the only transcription factor that had a canonical binding motif, known as octamer motif (ATGCA/TAAT), in all the eight HCC signature genes (Fig. 3a). Three of them (*TRIP13*, *CEP72*, *CHRM2*) had a single OCT4 binding motif while the other genes had multiple OCT4 binding motifs in their promoter regions. Based on this information, we selected *TK1* (multiple OCT4 binding motifs) and *TRIP13* (single OCT4 binding motif) as representatives for further in-depth validation and characterization. Our analysis additionally revealed that OCT4 binding motifs were present in the promoter regions of *TTK* [11] and *CDC20* [18] (Fig. S4), two HCC biomarkers reported in independent studies that also exhibited high T/P expression level ratios in the Huang et al. [10] study (Table S6). Thus, two HCC biomarker genes reported in the literature and eight HCC signature genes identified in this study all harbor canonical OCT4 binding motifs in their promoter regions, suggesting the possibility of common regulation of HCC signature genes by OCT4 at the transcriptional level.

Since our bioinformatics analysis revealed multiple or one putative OCT4 binding octamer motifs in the promoter regions of the *TK1* or *TRIP13* gene, respectively (Fig. 3a), we synthesized biotin-labeled DNA probes spanning the octamer motifs (Fig. 3b) and tested their binding with OCT4 using an electrophoretic mobility shift assay (EMSA). As the *NANOG* promoter contains a well-characterized octamer motif [15], a biotin-*NANOG* probe was taken as a positive control for all other probes tested. As expected, wild type recombinant OCT4 protein bound to the *NANOG* probe (Fig. S5A and S5B). It also bound three *TK1* probes with equivalent binding affinity to the *NANOG* probe, of which the *TK1* probe 2 bound most strongly (Fig. S5A). In addition, the *TRIP13* probe bound OCT4, albeit with a lower affinity than the *NANOG* probe (Fig. S5B). Having established a direct binding between OCT4 and the *TK1* and *TRIP13* promoter probes in vitro, we next tested such binding through EMSA using Hep3B cell nuclear extracts that contain endogenous OCT4 protein. We observed a band with both the *TK1* and the *TRIP13* probe that migrated at a similar position as the *NANOG* probe/recombinant OCT4 protein complex, indicating the presence of *TK1* probe/OCT4 and *TRIP13* probe/OCT4 complexes (Fig. 3c, arrow). In addition, multiple bands with both probes that migrated more slowly were noted (Fig. 3c, arrowheads). These may represent probes bound to larger protein complexes including OCT4. The intensities of the slow-migrating bands appeared to be stronger than that of the *TK1* probe/OCT4 band, and even stronger than that of the *TRIP13* probe/OCT4 band, suggesting that OCT4 in complex with other partners is likely the major form over the monomeric OCT4 form binding to the *TK1*/*TRIP13* promoters. Furthermore, we validated the *TK1*/*TRIP13* promoter-OCT4 interactions in Hep3B cells in vivo using a chromatin immunoprecipitation (ChIP) assay. We used a rabbit anti-OCT4 antibody to specifically precipitate OCT4-bound DNA fragments from Hep3B cell lysates, and quantified the OCT4-binding motif-containing DNA fragments of the *TK1* or *TRIP13* promoters using specific primers for qPCR. Compared to rabbit IgG that served as a negative control, we found that anti-OCT4 enriched the *TK1* and *TRIP13* promoter fragments by 2.5-3 fold (Fig. 3d), confirming bona fide binding between OCT4 and the *TK1*/*TRIP13* promoters in vivo.

**Fig. 4:**
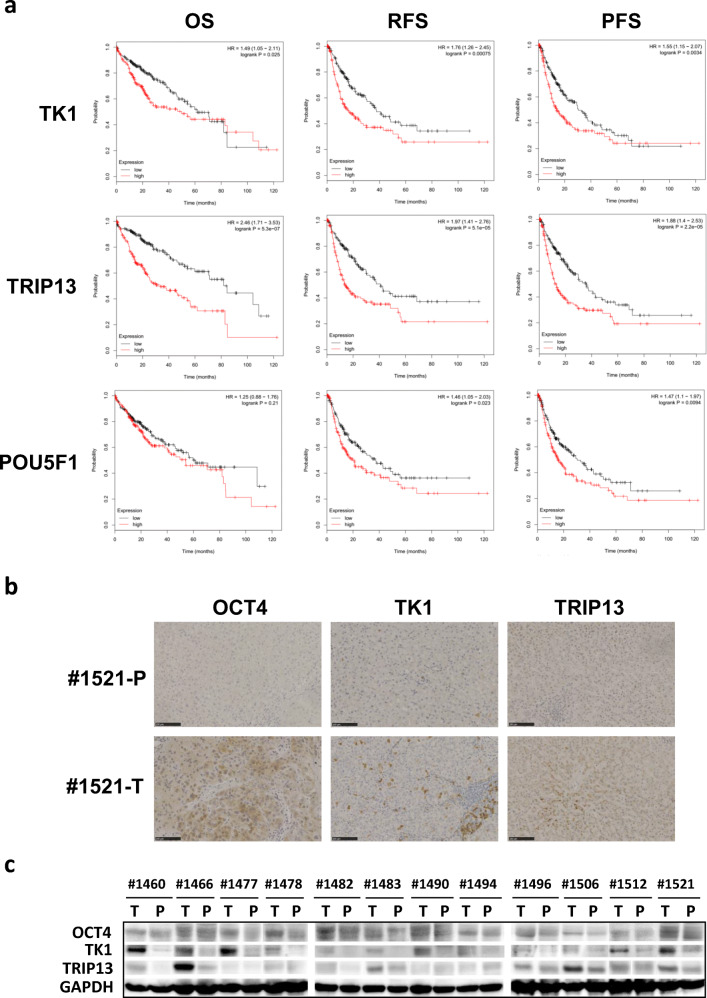
*TK1* and *TRIP13* expression correlates with OCT4 expression in HCC. **a** High *TK1*, *TRIP13* and *POU5F1* mRNA levels were associated with poor survival rates of HCC patients. Kaplan-Meier survival curves were analyzed as described in the Materials and methods section. The *p*-values of the *TK1* mRNA levels associated with overall survival (OS), relapse-free survival (RFS) and progression-free survival (PFS) were 0.025, 0.00075 and 0.0034, respectively. The *p*-values of the *TRIP13* mRNA levels associated with OS, RFS and PFS were 0.00000053, 0.000051 and 0.000022, respectively. The *p*-values of *POU5F1* mRNA levels associated with OS, RFS and PFS were 0.21, 0.023 and 0.0094, respectively. **b** IHC detection of OCT4, TK1 and TRIP13 in paired APT (P) and HCC (T) tissue samples from HCC patient #1521. OCT4 antibody: ab-109,183, diluted 1:500; TK1 antibody: ab-76,495, diluted 1:100; TRIP13 antibody: ab-204,331, diluted 1:100. Bars, 100 μm. **c** Western blotting of OCT4, TK1 and TRIP13 proteins in paired HCC (T) and APT (P) tissue samples from 12 individual HCC patients. GAPDH was used as a loading control

To correlate the expression levels of eight HCC signature genes with that of the *OCT4* gene (*POU5F1*) in tumor tissues versus APTs, we first examined the *OCT4* mRNA levels in HCC tumors (T) and APTs (P) by qRT-PCR using primers specifically amplifying a fragment of the OCT4A transcript [14]. Our results revealed higher levels of the OCT4A transcript in HCC tumors than in their paired APTs (Fig. S6). The RNA-seq data from Huang et al. [10], however, indicated no correlation or a slight negative correlation between *OCT4* and the HCC signature gene mRNA levels (Table S6). Nevertheless, we found that the mRNA levels of *TK1*, *TRIP13* and *POU5F1* in a cohort of 384 HCC samples from TCGA database were all negatively correlated with patient overall survival (OS), relapse-free survival (RFS) and progression-free survival (PFS) (Fig. 4a), strengthening the HCC biomarker status of *TK1* and *TRIP13* on the one hand, and implicating their association with OCT4 on the other hand. Further clinicopathological characterization of the 384 HCC samples (Table S2) revealed that, in general, high mRNA levels of most HCC signature genes correlated with the Ragnum Hypoxia Score (hypoxic ≥ 0.5), and that low mRNA levels of most HCC signature genes correlated with the Aneuploidy Score (= 0). High mRNA levels of *TK1*, *TRIP13* and *POU5F1* correlated with T3/T4 tumor stage. No overt correlations between the HCC signature gene mRNA levels and other clinicopathological parameters (such as Mutation Count, Cancer Metastasis Stage Code, Cancer Publication Version Type, Sex, Diagnosis Age, Neoplasm Histologic Grade) were noted.

Since mRNA levels may be discordant with protein levels for genes expressed in the context of HCC [19], we next took *TK1* and *TRIP13* as representatives for the eight HCC signature genes and also examined their protein levels relative to OCT4 protein levels by immunohistochemistry and Western blotting. First, we compared the protein expression levels and the localization of OCT4 in HCC tumor tissues versus APTs. Using immunohistochemistry, we found that OCT4 was expressed in both the cytoplasm and nucleus of the liver cells in HCC tissues but not in APTs (Fig. 4b). The cytoplasmic staining of OCT4 was relatively even among most HCC cells, while the nuclear staining was mainly confined to a few cells. A similar heterogeneous expression and predominant cytoplasmic staining pattern was also seen with TK1 and TRIP13 in tumor tissues, but not in APTs (Fig. 4b). Higher protein levels of OCT4, TK1 and TRIP13 in HCC tumor tissues over APTs were confirmed in 12 pairs of matched samples by Western blotting (Fig. 4c). Overall, the correlation between OCT4 protein level and TK1 protein level was higher than that between OCT4 and TRIP13. Collectively, the concurrent higher expression of OCT4, TK1 and TRIP13 in HCC tumor tissues compared to APTs not only supports roles of TK1 and TRIP13 as novel HCC biomarkers, but also underscores the hypothesis that OCT4 may be a common transcription factor for TK1 and TRIP13 in the context of HCC.

### Validation of a crucial role of OCT4 in HCC tumorigenesis

**Fig. 5:**
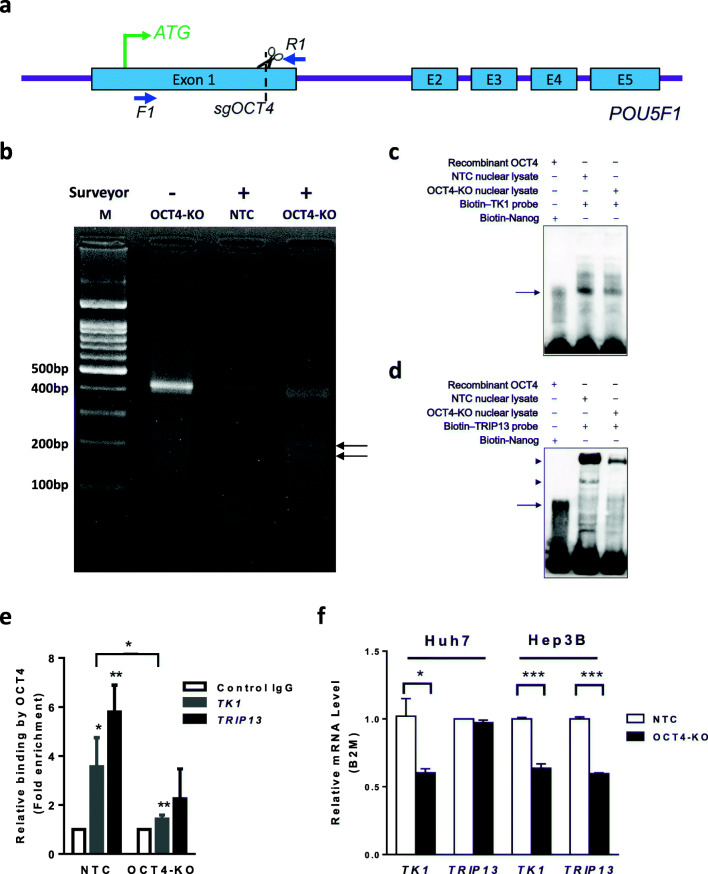
OCT4 KO reduces promoter occupancy and transcriptional activation of the *TK1* and *TRIP13* genes by OCT4. **a** Schematic representation of OCT4 knockout (KO) mediated by CRISPR/Cas9. **b** Surveyor assays with the OCT4 KO cell lines and NTC cell line showing obvious cleavage of OCT4 PCR products spanning the Cas9 target site, indicating a high probability of DNA indel formation and, thus, KO. Arrows indicate surveyor nuclease cleaved DNA fragments. **c**, **d** EMSA of biotinylated *TK1* (**c**) or *TRIP13* (**d**) probes incubated with nuclear lysates of NTC or OCT4-KO Huh7 cells and, as a positive control, EMSA of biotinylated *NANOG* probes incubated with recombinant human OCT4 proteins. The arrows indicate complexes formed between the biotinylated probes and monomeric OCT4 proteins, while the arrowheads indicate the biotinylated probes bound with larger protein complexes including OCT4. The data shown are from one of three independent experiments which yielded similar results. **e** NTC or OCT4-KO Huh7 DNAs were chemically cross-linked, sonicated and immunoprecipitation with 5 μg rabbit anti-OCT4 (ab 19,857) and 100 μl protein A/G agarose beads (Pierce 20,421), with normal rabbit IgG serving as negative control. Anti-OCT4 precipitated DNA fragments corresponding to the *TK1* or *TRIP13* genes were quantified using qPCR and expressed as fold enrichment over anti-rabbit IgG precipitated DNA fragments (Control IgG). The data are expressed as mean ± S.D. of triplicate measurements from one of three independent experiments, which yielded similar results. *p < 0.05, **p < 0.01. **f** Lentivirus-infected NTC or OCT4-KO Huh7/Hep3B cells were selected in puromycin for 7 days, expanded for another 7 days and harvested. *TK1* and *TRIP13* transcription was quantified by qRT-PCR with *B2M* as internal control. The data are expressed as mean ± SD. *p < 0.05, ***p < 0.001

To conclusively determine whether OCT4 serves as a common transcription factor for the newly-identified HCC signature/biomarker genes, and whether it is required for the tumorigenesis of HCC cells, the *POU5F1* gene was knocked out (KO) in Hep3B and Huh7 cells through CRISPR/Cas9-mediated gene editing (Fig. 5a) as previously reported [14]. Successful KO was demonstrated by cleavage of *OCT4* PCR products spanning the Cas9 target site using Surveyor nuclease (Fig. 5b). Next, we compared the binding of endogenous OCT4 to the *TK1*/*TRIP13* promoters in NTC versus OCT4-KO Huh7 cells. Using EMSA, we observed a significant reduction in monomeric OCT4-bound *TK1* promoter fragments (Fig. 5c, arrow) and complexed OCT4-bound *TRIP13* promoter fragments (Fig. 5d, arrowheads) in OCT4-KO cells, which were consistent and corroborated by ChIP data (Fig. 5e). Accordingly, transcription of the *TK1* gene was found to be reduced in OCT4-KO Huh7 cells and of both genes in OCT4-KO Hep3B cells (Fig. 5f). Taken together, our results indicate that OCT4 in HCC cells acts as a common transcription factor for several novel HCC signature genes identified in this study.

**Fig. 6:**
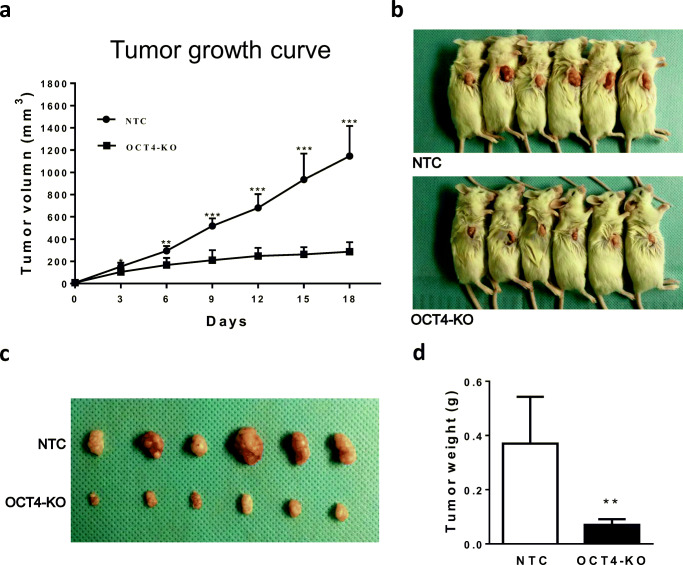
OCT4 KO effectively suppresses HCC tumor growth in nude mice. **a** Tumor growth curve: the mice were randomly divided after which OCT4-KO or NTC Huh7 cells (2 × 10^6^) were inoculated subcutaneously into each mouse. The diameters of the tumors were measured every three days using precision calipers. The tumor mass (xenograft) volumes were calculated with the formula: volume = [(tumor length) × (tumor width)^2^]/2. (b-d) 18 days after inoculation the mice were sacrificed (**b**) after which the tumors removed, photographed (**c**) and weighed (**d**). The data are expressed as mean ± SD. *p < 0.05, **p < 0.01, ***p < 0.001

To examine the effect of OCT4 KO on the tumorigenicity of HCC cells in an in vivo setting, equal numbers of OCT4-KO or NTC Huh7 cells were inoculated subcutaneously into nude mice, after which the volumes of the xenografted tumors were recorded every 3 days. The weights of the tumors were measured after 18 days when the mice were sacrificed. It was evident that the xenografted tumors grew rapidly and steadily in the NTC group, but significantly slower in the OCT4-KO group (Fig. 6a). At day 18 post inoculation, the differences in tumor volumes (Fig. 6b, c) and tumor weights (Fig. 6d) between the two groups were prominent, substantiating a crucial role of OCT4 in the tumorigenesis of HCC cells.

## Discussion

HCC represents > 90% of primary liver cancers and is a major health problem due to its poor prognosis [8, 20]. A number of risk factors for the development of HCC has been identified, and over 50% of HCC cases are attributable to persistent HBV infections [5]. The incidence of HCC is growing worldwide, especially in less developed regions, which suffer from more HBV infections [21]. HBV-mediated hepatocarcinogenesis is a multistep process that involves complex interactions between viral components, host genetic and environmental factors [5, 21]. To improve the prognosis of HBV-related HCC, it is important to identify molecular biomarkers and signature genes that act at different stages of HCC development.

RNA-seq is a recently-developed approach based on deep-sequencing technologies that can be utilized for genome-wide transcriptome profiling. It provides a more precise measurement of gene transcript levels and their isoforms than other available methods [22]. Here, we identified 164 DEGs in HCC tumor tissues compared to their matched APTs. Confirmation of the RNA-seq data for 14 selected DEGs using qRT-PCR revealed a high correlation between the two measurements. Moreover, the eight HCC signature gene RNA-seq measurements in our study highly correlated with those reported by Huang et al. [10], although the identified DEGs were not further filtered and ranked in the latter study. Such a high correlation indicates a high robustness and reproducibility of the experimental systems used in both studies.

Some of the eight newly-identified HCC signature genes have previously been related to the development of various human cancers including HCC. First, overexpression of *CTTN* (cortactin) has been closely associated with a poor prognosis in HCC resulting from an increased cell motility and metastasis [23, 24]. Second, upregulation of *FTH1* (ferritin heavy chain 1) in HCC cells by TNF-α has been found to attenuate starvation-induced apoptosis [25]. Third, *TK1* (thymidine kinase-1) has been associated with the early development of breast, lung, heart, esophageal and gallbladder cancer [26–29]. A clinical investigation showed that serum TK1 levels > 2.0 pmol/L may indicate an increased risk for the development of malignancies later in life [30]. The TK1 serum levels in patients with HCC were significantly higher than those in patients with benign hepatic diseases, placing serum TK1 as a complementary biomarker for the diagnosis of HCC [31]. Fourth, systematic analysis of data from the Gene Expression Omnibus (GEO) database revealed that *TRIP13* (thyroid hormone receptor interactor 13) was upregulated in 12 human cancers, and that a high *TRIP13* expression indicated a poor prognosis for patients with liver, breast, gastric and lung cancer [32]. *TRIP13* gene copy numbers have been found to be increased in early-stage non-small-cell-lung cancer [33], and increased *TRIP13* transcript and protein levels have been correlated with prostate cancer progression [34]. Overexpression of *TRIP13* in non-malignant fibroblasts resulted in malignant transformation, and high expression of *TRIP13* in head and neck cancer cells led to aggressive, treatment-resistant tumors and enhanced DNA damage repair via nonhomologous end joining [35]. Fifth, the expression of *AMBP* has been found to be down-regulated in both HCC tissues and cell lines [36], a finding consistent with our current results. As yet, the involvement of the remaining three genes (*CEP72*, *FLAD1*, *CHRM2*) in hepatocarcinogenesis requires further validation.

Our data indicate that OCT4 may serve as a potential common transcription factor for all the eight HCC signature genes identified in this study. OCT4, encoded by the *POU5F1* gene, is a member of the POU family of transcription factors that is abundantly expressed in pluripotent stem cells (such as embryonic stem cells, embryonal carcinoma cells, induced pluripotent stem cells) and plays an essential role in the early development of mammalian embryos. The POU domains of the OCT4 protein can independently and flexibly bind half-sites of the characteristic octamer motif (ATGCA/TAAT) through which OCT4 recognizes enhancer or promoter regions of hundreds target genes [37]. This flexibility allows OCT4 to form heterodimers with other transcription factors such as SOX2, cooperatively activating pluripotency and self-renewal-associated genes while simultaneously repressing genes that promote differentiation [38]. Similar to, but distinct from its well characterized roles in pluripotent stem cells, OCT4 is generally considered to promote the self-renewal, survival, metastasis and drug resistance of cancer stem cells (CSCs) [39–41].

Conditions within the microenvironment of HBV-infected cells may play an important role in the programming of liver cells towards cancer stem cells (CSCs) and the divergence of multiple HBV-induced pathways towards epithelial-mesenchymal transition (EMT) or stemness [42]. IL-6 is secreted by inflammatory and stromal cells during liver regeneration and is known to support the conversion of non-CSCs to CSCs [43]. Chang et al. [44] found that HBV-related HCC patients had a higher serum level of IL-6, leading to increased expression of autocrine insulin-like growth factor I (IGF-I) and IGF-I receptor (IGF-IR), which subsequently promote the expression of OCT4 and NANOG in a STAT3-dependent manner. They further found that inflammation-conditioned medium generated by lipopolysaccharide-stimulated U937 human leukemia cells significantly up-regulated the expression of OCT4/NANOG, IGF-I/IGF-IR and activated IGF-IR/AKT signaling in HBV-active (HBV + HBsAg+) HCC cells [45]. The close association between IL-6/IGF-1R signaling and HBV-related HCC progression suggests that HBV and proinflammatory cytokines are both required for and collaboratively involved in pluripotency factor induction and CSC formation, but the exact mechanism inducing OCT4 re-expression remains to be elucidated.

HBx of the HBV viral component is a multifunctional protein that activates many viral and cellular genes, modulating multiple cellular signaling pathways and regulating host cell proliferation, apoptosis and invasion [5]. HBx does not bind DNA directly but regulates gene expression by transactivating multiple transcription factors. So far, there is no evidence that HBx can directly interact with the OCT4 protein, but there are several reports showing that it can up-regulate OCT4 expression in HCC cells via various routes. Arzumanyan et al. [46] reported that overexpression of HBx in HepG2 cells may be associated with enhanced expression of the pluripotency factors OCT4, NANOG and KLF4, and the stemness-associated markers EpCAM and β-catenin. Another study showed that HBV viral components including HBx may induce regulated intramembrane proteolysis (RIP) of EpCAM in HCC cells, and cleavage of the EpCAM/β-catenin complex translocated to the nucleus leading to activation of canonical Wnt signaling that is accompanied with up-regulated OCT4 expression [47]. There is evidence that β-catenin can bind specifically and directly to the promoter of OCT4 in mouse embryonic stem cells [48], but such a direct regulation has not been demonstrated in CSCs. Recently, HBx has been shown to stimulate AFP expression prior to up-regulation of pluripotency factors such as OCT4, leading to partial reprogramming of liver cells toward HCC progenitor/stem cells [49]. Furthermore, HBx can activate FOXM1 expression in HCC cells via the ERK/CREB pathway [50], and FOXM1 has been reported to directly bind to and activate the OCT4 promoter in P19 embryonal carcinoma cells [51]. Independently, HBV PreS1 has been found to stimulate the expression of multiple pluripotency factors including OCT4 and the self-renewal of liver CSCs [52]. However, none of the above studies has definitively identified factors/mechanisms directly linking HBx to up-regulated OCT4 expression in the context of HBV-related HCC. In a study aimed at identifying differentially-expressed transcription factors between CSCs and non-CSCs from HCC cell lines, ZIC2 was found to be highly expressed in CSCs [16]. Additional studies demonstrated that it acts as a direct transcription factor for OCT4. It remains to be established, however, whether this also holds in the context of HBV-related HCC and whether/how HBV viral components play a role. Interestingly, the extent of increase in OCT4 mRNA levels in HCC tumor tissues was found to be significantly lower than that in OCT4 protein levels, consistent with what we found in this study.

Taken together, we identified multiple HCC signature genes that may serve as biomarkers for HBV-related HCC diagnosis and prognosis. Their common transcriptional regulation by OCT4 implicates a key regulatory role of OCT4 in the occurrence and development of HCC, and thus positions it as a potential drug target for HCC.

## Electronic supplementary material

ESM 1 (DOC 2683 kb)

## Abbreviations

APT: Adjacent peritumor tissue
BP: Biological process
CC: Cellular compound
ChIP: Chromatin immunoprecipitation
DEG: Differentially expressed gene
EMSA: Electrophoretic mobility shift assay
FDR: False discovery rate
FPKM: Fragments per kilobase million
GO: Gene ontology
HBV: Hepatitis B virus
HCC: Hepatocellular carcinoma
HCV: Hepatitis C virus
KO: Knockout
IHC: Immunohistochemistry;
MF: Molecular function
NTC: Non-targeting control
qRT-PCR: quantitative real-time PCR
RPKM: Reads per kilobase million
RNA-seq: RNA sequencing
