# Supplementary material for: Multiple novel hepatocellular carcinoma signature genes are commonly controlled by the master pluripotency factor OCT4
Source: Cell Oncol (Dordr). 2019 Dec 17;43(2):279–95. doi: 10.1007/s13402-019-00487-3 (PMC12990707; doi:10.1007/s13402-019-00487-3)
Supplement: Supplementary file 1 — (DOC 2683 kb) [file 13402_2019_487_MOESM1_ESM.doc]

**SUPPLEMENTARY DATA**

**Multiple novel hepatocellular carcinoma signature genes are transcriptionally controlled in common by the master pluripotency factor OCT4**

Chao Ye, Xiaoqian Zhang, Xinyu Chen, Qingyi Cao, Xiaobing Zhang, Yanwen Zhou, Wenxin Li, Liangjie Hong, Haiyang Xie, Xiaoli Liu, Hongcui Cao, Ying-Jie Wang* and Bo Kang*

**Inventory**

**Supplemental Data**

**Figure S1.** The coverage of all the gene sequencing in three paired samples.

**Figure S2.** Expression levels of identified DEGs in 10 paired HCC samples in the literature.

**Figure S3.** Expression levels of eight DEGs in 10 paired HCC tissues (Tumor) and APTs (APT) in the literature.

**Figure S4.** OCT4 binding motifs present at the promoter regions of TTK and CDC20.

**Figure S5.** Validation of biotinylated TK1 and TRIP13 probes for EMSA.

**Figure S6.** Detection of OCT4A transcript in HCC tumors by RT-PCR.

**Table S1.** Primer sequences for RT-PCR.

**Table S2.** Correlations of eight DEGs and POU5F1 expression level with clinicopathological characteristics in 348 HCC clinical samples from TCGA database.

**Table S3.** Summary of overall characteristics of RNA-Seq data.

**Table S4.** Mapped gene numbers of each paired tumor and APT.

**Table S5.** Gene ontology for functional enrichment analysis for DEGs.

**Table S6.** The RPKM value of each gene for 10 paired samples.

**
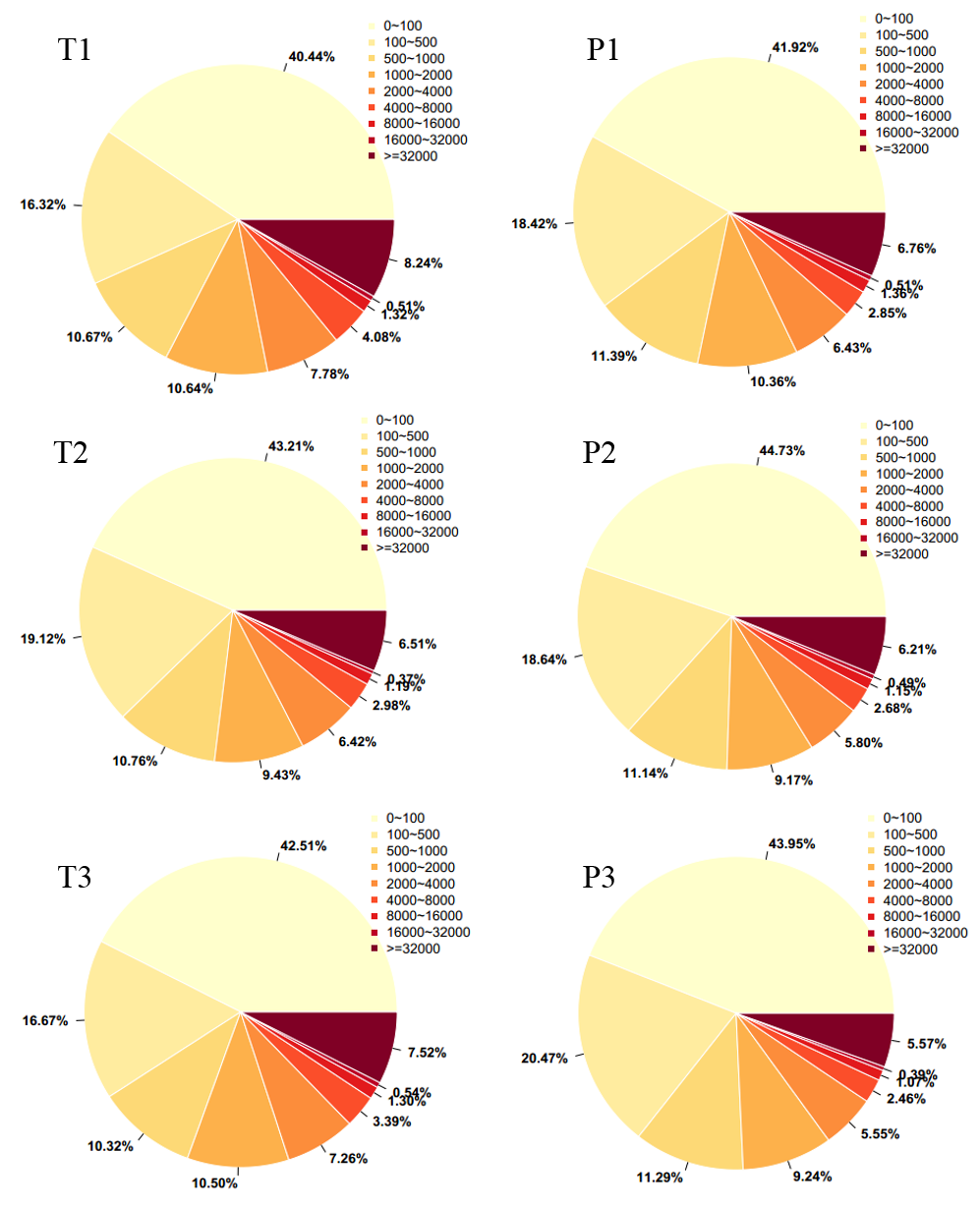
**

**Figure S1. The coverage of all the gene sequencing in three paired samples.**


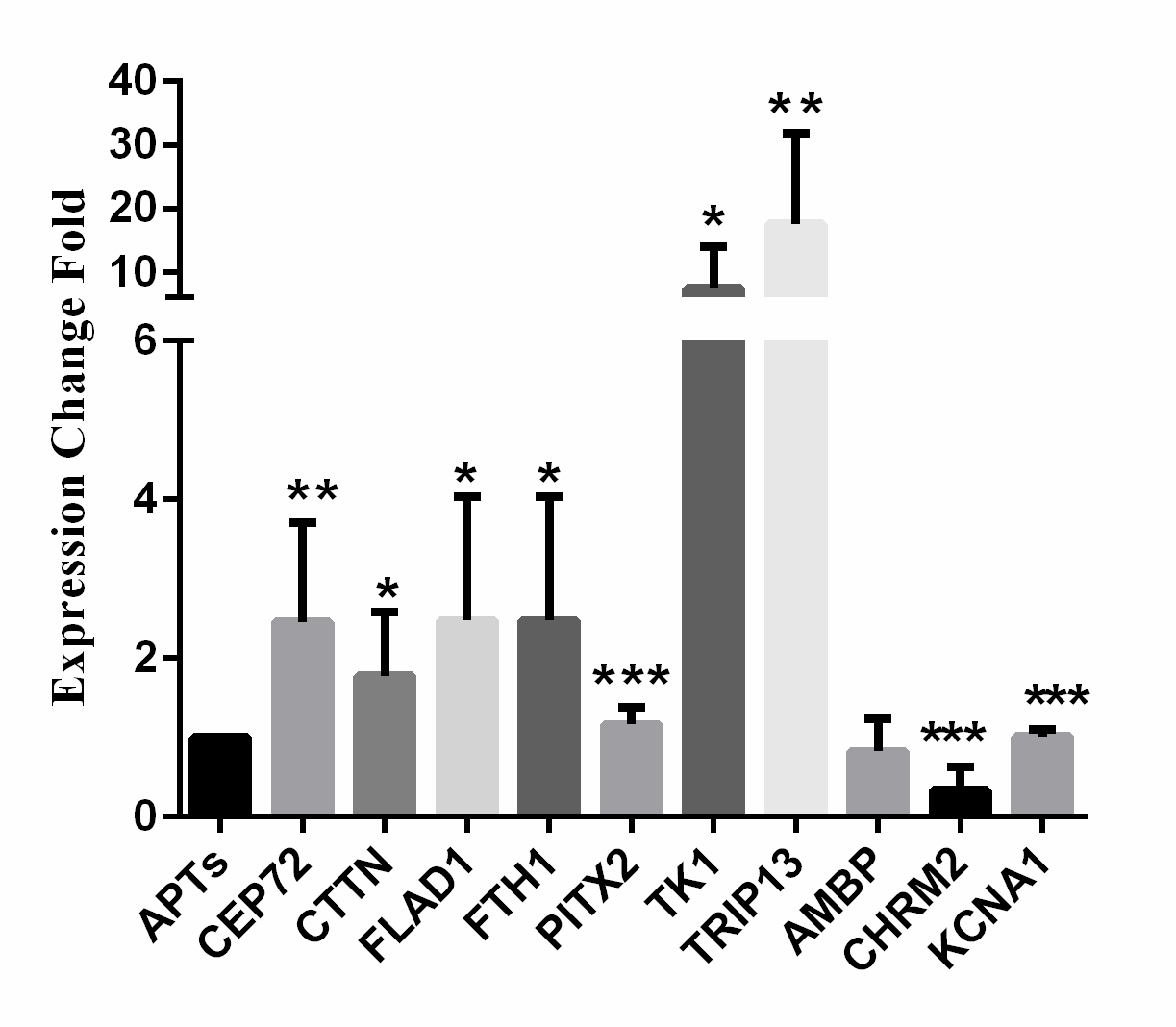


**Figure S2. Expression levels of identified DEGs in 10 paired HCC samples in the literature.** The data were retrieved from Reference [10], Table S3. The tumor/peritumor ratios (T/P) of 10 pairs of matched samples were calculated and plotted as fold changes of expression levels for the identified 10 DEGs. * p<0.05, **p<0.01, ***p<0.001.

###

**Figure S3. Expression levels of eight DEGs in 10 paired HCC tissues (Tumor) and APTs (APT) in the literature.** The data were retrieved from Reference [10], Table S3.

###

###

**Figure S4. OCT4 binding motifs present at the promoter regions of TTK and CDC20.**

**Figure S5. Validation of biotinylated TK1 and TRIP13 probes for EMSA.** EMSA of three biotinylated TK1 probes (A), one biotinylated TRIP13 probe (B), and one biotinylated NANOG probe (as positive control) incubated with recombinant human OCT4 proteins. The arrow indicates complexes formed between the biotinylated probes and monomeric OCT4 proteins.

**Figure S6. Detection of OCT4A transcript in HCC tumors by RT-PCR.** The OCT4 mRNA levels in three paired HCC tumors (T) and APTs (P) were qualitatively assessed by RT-PCR using primers specifically amplifying a 128 bp fragment of the OCT4A transcript.

**Table S1. Primer sequences for RT-PCR**

| **Gene symbol** | **NCBI Gene ID** | **Forward primer (5’-3’)** | **Reverse Primer (3’-5’)** | **Size of product**  **(bp)** |
| --- | --- | --- | --- | --- |
| CEP72 | 55722 | TTCGGCTCCACGCCTTAAC | GGCGGTAGTCAGGCTCAAC | 81 |
| CTTN | 2017 | GCTTTGAGTATCAAGGCAAAACG | CCAAGGGCACATTTGTCTTGT | 112 |
| FLAD1 | 80308 | TATGGCACAGATCCTTGCACT | TGGGAAGAGGTAGACGTTTCG | 75 |
| FTH1 | 2495 | CCCCCATTTGTGTGACTTCAT | GCCCGAGGCTTAGCTTTCATT | 180 |
| MMP12 | 4321 | GGAATCCTAGCCCATGCTTTT | CATTACGGCCTTTGGATCACT | 174 |
| PITX2 | 5308 | TGTGGACCAACCTTACGGAAG | ATGAGCCCATTGAACTGCGG | 133 |
| TK1 | 7083 | GGGCAGATCCAGGTGATTCTC | TGTAGCGAGTGTCTTTGGCATA | 130 |
| TRIP13 | 9319 | TGTGTAAAGCGTTAGCCCAGA | GCCACTTTCCGAAAACCACTTA | 116 |
| ZNF695 | 57116 | ATCTCCCTTGGTGAGGATAGC | GACAAAACTGAGTGTTTGGCTG | 146 |
| AMBP | 259 | CTCTCGGATCTATGGGAAGTGG | CGTGCTCACTGTCATCCTGTC | 91 |
| CHRM2 | 1129 | AACTCCTCTAACAATAGCCTGGC | GTTCCCGATAATGGTCACCAAA | 108 |
| C14orf180 | 400258 | AGGGAGGACAACAGGAAGTG | CAGCAATGTAGTGGACGGTCA | 142 |
| KCNA1 | 3736 | TAGTGCAGTGTACTTTGCCGA | GTCACCGTATCCTACAGTGGT | 109 |
| PRB2 | 653247 | CCACAAGGAGACAACAAGTCC | AGGAGATCGAGAACTTCGGGA | 225 |
| hGAPDH* | 2597 | CTCTCTGCTCCTCCTGTTCG | ACGACCAAATCCGTTGACTC | 112 |

*hGAPDH was used as the reference.

**Table S2. Correlations of eight DEGs and POU5F1 expression level with clinicopathological characteristics in 348 HCC clinical samples from TCGA database**

**Table S3. Summary of overall characteristics of RNA-Seq data**

| **Sample** | **Total reads (PE)** | **Total mapped reads** | **Mapped pairs** |
| --- | --- | --- | --- |
| T1 | 53.5M | 58.4M (54.6%) | 22.5M (42.0%) |
| P1 | 36.7M | 60.2M (82.0%) | 25.2M (68.7%) |
| T2 | 41.6M | 59.9M (72.0%) | 25.0M (60.0%) |
| P2 | 48.0M | 77.6M (80.8%) | 32.5M (67.6%) |
| T3 | 49.7M | 68.4M (68.8%) | 28.5M (57.3%) |
| P3 | 47.2M | 72.9M (77.3%) | 30.4M (64.4%) |

**Table S4. Mapped gene numbers of each paired tumor and APT**

| **Sample** | **Up-regulated genes** | **Down-regulated genes** |
| --- | --- | --- |
| T1/P1 | 12063 | 8128 |
| T2/P2 | 13078 | 6921 |
| T3/P3 | 11080 | 9237 |

**Table S5. Gene ontology for functional enrichment analysis for DEGs**

| **label** | **id** | **Gene number** | **Enrichment** | **P value** |
| --- | --- | --- | --- | --- |
| **GO_ BP*** | | | | |
| system process | GO:0003008 | 23 | C=1695; O=23; E=10.31; R=2.23; rawP=0.0001 | 0.0204 |
| endocrine system development | GO:0035270 | 6 | C=136; O=6; E=0.83; R=7.25; rawP=0.0002 | 0.0244 |
| embryonic organ morphogenesis | GO:0048562 | 7 | C=217; O=7; E=1.32; R=5.30; rawP=0.0004 | 0.0244 |
| epithelial cell proliferation | GO:0050673 | 7 | C=233; O=7; E=1.42; R=4.94; rawP=0.0005 | 0.0278 |
| response to radiation | GO:0009314 | 8 | C=319; O=8; E=1.94; R=4.12; rawP=0.0007 | 0.0329 |
| regulation of cellular process | GO:0050794 | 62 | C=7687; O=62; E=46.76; R=1.33; rawP=0.0007 | 0.0329 |
| G-protein coupled receptor signaling pathway | GO:0007186 | 11 | C=606; O=11; E=3.69; R=2.98; rawP=0.0011 | 0.0336 |
| neurological system process | GO:0050877 | 17 | C=1237; O=17; E=7.53; R=2.26; rawP=0.0011 | 0.0336 |
| regulation of protein secretion | GO:0050708 | 5 | C=124; O=5; E=0.75; R=6.63; rawP=0.0009 | 0.0336 |
| regulation of epithelial cell proliferation | GO:0050678 | 6 | C=200; O=6; E=1.22; R=4.93; rawP=0.0014 | 0.0407 |
| **GO_ MF** | | | | |
| neurotransmitter receptor activity | GO:0030594 | 6 | C=73; O=6; E=0.44; R=13.65; rawP=4.97e-06 | 0.0005 |
| **GO_CC*** | | | | |
| extracellular region | GO:0005576 | 28 | C=2140; O=28; E=13.43; R=2.09; rawP=9.94e-05 | 0.0044 |
| very-low-density lipoprotein particle | GO:0034361 | 3 | C=20; O=3; E=0.13; R=23.91; rawP=0.0003 | 0.0066 |
| triglyceride-rich lipoprotein particle | GO:0034385 | 3 | C=20; O=3; E=0.13; R=23.91; rawP=0.0003 | 0.0066 |
| high-density lipoprotein particle | GO:0034364 | 3 | C=25; O=3; E=0.16; R=19.12; rawP=0.0005 | 0.0088 |
| protein-lipid complex | GO:0032994 | 3 | C=38; O=3; E=0.24; R=12.58; rawP=0.0017 | 0.0187 |
| synaptic membrane | GO:0097060 | 6 | C=215; O=6; E=1.35; R=4.45; rawP=0.0024 | 0.0235 |
| integral to plasma membrane | GO:0005887 | 16 | C=1220; O=16; E=7.65; R=2.09; rawP=0.0038 | 0.0304 |
| extracellular region part | GO:0044421 | 15 | C=1099; O=15; E=6.90; R=2.18; rawP=0.0035 | 0.0304 |
| intrinsic to plasma membrane | GO:0031226 | 16 | C=1264; O=16; E=7.93; R=2.02; rawP=0.0054 | 0.0396 |
| extracellular space | GO:0005615 | 12 | C=856; O=12; E=5.37; R=2.23; rawP=0.0074 | 0.0465 |

BP: biological process; CC: cellular compound; MF: molecular function. *top 10 labels of biological process (BP) and cellular compound (CC)

**Table S6. The RPKM value of each gene for 10 paired samples**


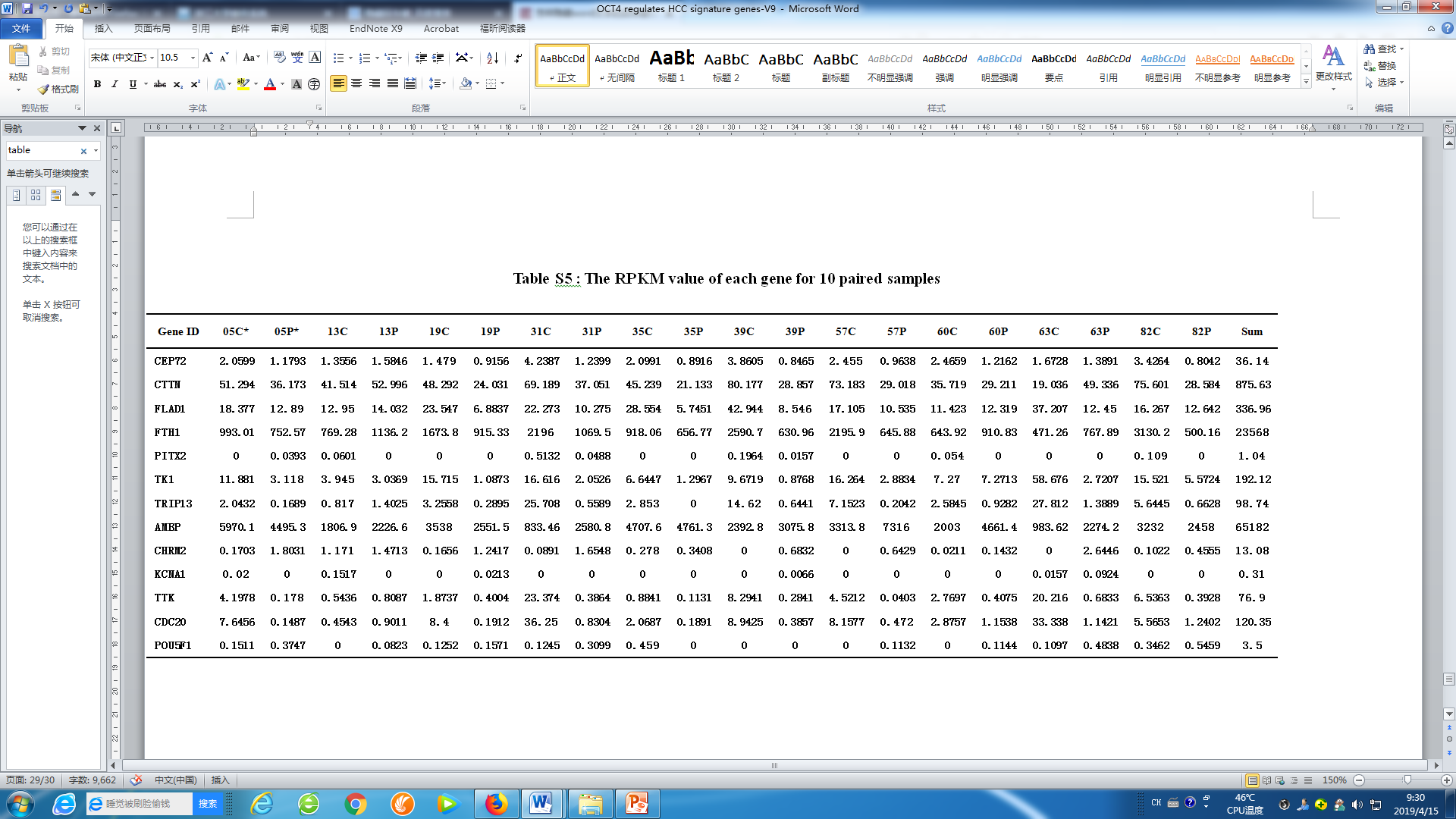


The data were retrieved from Reference [10], Table S3.
